# Carpal Tunnel Syndrome: A Review of Literature

**DOI:** 10.7759/cureus.7333

**Published:** 2020-03-19

**Authors:** Alessia Genova, Olivia Dix, Asem Saefan, Mala Thakur, Abbas Hassan

**Affiliations:** 1 Internal Medicine, Xavier University School of Medicine, Oranjestad, ABW; 2 Plastic and Reconstructive Surgery, Northwestern University Feinberg School of Medicine, Chicago, USA

**Keywords:** carpal tunnel syndrome, cts syndrome, diagnostic tools, clinical features, management, carpal tunnel release

## Abstract

Carpal tunnel syndrome (CTS) is a common medical condition that remains one of the most frequently reported forms of median nerve compression. CTS occurs when the median nerve is squeezed or compressed as it travels through the wrist. The syndrome is characterized by pain in the hand, numbness, and tingling in the distribution of the median nerve. Risk factors for CTS include obesity, monotonous wrist activity, pregnancy, genetic heredity, and rheumatoid inflammation. The diagnosis of CTS is conducted through medical assessments and electrophysiological testing, although idiopathic CTS is the most typical method of diagnosis for patients suffering from these symptoms. The pathophysiology of CTS involves a combination of mechanical trauma, increased pressure, and ischemic damage to the median nerve within the carpal tunnel. The diagnosis of CTS patients requires the respective medical professional to develop a case history associated with the characteristic signs of CTS. In addition, the doctor may question whether the patients use vibratory objects for their tasks, the parts of the arm where the sensations are felt, or if the patient may already have predisposing factors for CTS incidence. During the diagnosis of CTS, it is essential to note that other conditions may also provide similar symptoms to CTS, thus requiring vigorous diagnosis to assert the medical condition of the patients. Doctors use both non-surgical and surgical treatments when addressing CTS. Non-surgical treatments include wrist splinting, change of working position, medications, and the use of alternative non-vibrating equipment at work. On the other hand, surgical methods include open release and endoscopic surgeries. This review of literature has provided an overview of CTS with an emphasis on anatomy, epidemiology, risk factors, pathophysiology, stages of CTS, diagnosis, and management options.

## Introduction and background

Carpal tunnel syndrome (CTS) is a common medical condition, which causes pain, numbness, and tingling in the hand and arm of the affected individual. CTS occurs when the median nerve is squeezed or compressed as it travels through the wrist. Risk factors for CTS include obesity, monotonous wrist activity, pregnancy, genetic heredity, and rheumatoid inflammation [1]. The symptoms for CTS may vary across patients. As such, they are classified differently into mild, moderate, and severe. The syndrome is characterized by pain in the hand, numbness, and tingling in the distribution of the median nerve. These sensations may be felt in the thumb, index finger, middle finger, and the radial side of the ring finger [2]. The painful feelings may result in a reduction in grip strength and hand function. The occurrence of CTS over a long time may also result in the muscles at the base of the thumb wasting away. An estimated 4% and 5% of people suffer from CTS worldwide, with the most susceptible population being elderly individuals aged between 40 and 60 years [3]. CTS is also more prevalent amongst women as compared to men. For instance, the UK General Practice Research Database in 2000 evaluated that CTS prevalence was 88 per 100,000 in males, while in women, the incidence was 193 per 100,000 [2]. More frequent evaluations of the incidence of CTS notes its occurrence to be higher for women aged between 45 and 54 years, while the risk is higher for men aged between 75 and 84 years [4]. CTS is a musculoskeletal disorder associated with work activity in the affected individuals, which is caused by strain and repetitive activity, making it a common problem across manual laborers. As such, CTS can also be associated with increased absences from work and further healthcare risks. This review article discusses the anatomy, epidemiology, risk factors, pathophysiology, stages, diagnosis, and management options of CTS.

## Review

Anatomy

The symptoms for CTS may tend to vary, which is the result of the variation in the anatomy. For instance, for the anatomical differences in the nerves, a bifid median nerve resulting from the high division is noted in 1% to 3.3% of the cases [3,5]. This is associated with the tenacity of the median artery or with an additional division of the superficial flexor of the third finger. Another variation is noted in the motor branch of the median nerve. In this variation, there are five types of starting points and paths of the thenar division. The most frequent type of variation is the extraligamentous form, which assumes 46% of the cases, while the subligamentous form accounts for 31%, and the transligamentous form takes 23% of the cases [3,6]. The nerve bundles intended for the thenar branch may be situated on the radial, anterior, or central part of the median nerve. In other instances, the thenar branch passes through a tunnel before entering the thenar muscles. These differences illustrate the inconstant motor effect in cases of severe compression on the median nerve. Another variation occurs in the palmar cutaneous branch of the median nerve. In this respect, the palmar cutaneous division often starts from 4 cm to 7 cm above the wrist fold and moves along near the median nerve for 1.6 to 2.5 cm^3^. The branch then enters a tunnel formed by the fascia at the medial edge of the flexor carpi radialis (FCR) and emerges 0.8 cm above the wrist flexion wrinkle, to innervate the skin of the thenar eminence. The palmar cutaneous branch may either go to the ulnar side of the median nerve or cross the transverse ligament of the carpus. Another variation, though rare, is the intratunnel positioning of the ulnar nerve. In the event of its occurrence, however, the irregularity shows the combined symptoms of the median and ulnar nerves [7]. Activities of the wrist joint also influence the form and size of the CT. During the normal range of wrist motion, the width of the tunnel decreases considerably, with the carpal bones moving relative to each other because of the bony walls of the tunnel being flaccid [8]. Flexion and extension also cause an increase in CT pressure. Conversely, the cross-section of the proximal opening of the CT decreases with the flexing of the wrist joint. This is because of the circular changes of the transverse carpal ligament (TCL) and the movement of the distal end of the capitates bone. Extreme extension causes the lunate bone to scrunch the passage while being pushed towards the interior part of the tunnel. The TCL is the dense, diminutive, and broad essential element of the flexor retinaculum (FR), ranging between 2 mm to 4 mm in thickness, an average width of 25 mm, and 31 mm in length [8-9]. It is a firm band, which forms from interwoven bundles of fibrous connective tissues. It also spreads from the distal part of the radius to the distal segment of the base of the third metacarpal. The average closeness to the central portion is 11 mm away from the capitate-lunate joint, while the average distal limit of the distal portion is 10 mm distal to the carpometacarpal joint of the third metacarpal [8].

Epidemiology

CTS is the most common entrapment condition affecting one or more peripheral nerves and resulting in numbness or weakness in the affected body organ. On average, at least 3.8% of people who complain of aching, unresponsiveness, and an itchy feeling in their hands have CTS [10-11]. Diagnosis for CTS is conducted through medical assessments and electrophysiological testing, although idiopathic CTS is the most typical method of diagnosis for patients suffering from these symptoms. In addition, the events of CTS occurrence occur at a rate of 276 per 100,000 annual reports, with the incidence rates being 9.2% for women and 6% in men [10,12]. Although CTS incidences are common across all age groups, it is more prevalent for adults between the age of 40 and 60 years. In regions like the United Kingdom, CTS occurrence is between 7%-16%, which is relatively higher as compared to the 5% incidence rates in the United States [13-14]. Most western nations indicate a rise in the number of work-related musculoskeletal disorders (WMSDs). This is associated with increased strain and repetitive movements by individuals. Europe, in 1998, for instance, reported more than 60% of upper limb musculoskeletal disorders recognized as work-related being CTS incidences [10]. The prevalence levels may also vary across the different occupations and industries, with industries, such as the fish processing industries reporting the occurrence of CTS in their workers estimated at 73% [10]. These views on the occurrence rates of CTS illustrate the weight of the challenge, making it a significant area of concern, which would require effective strategies for management.

Risk factors

Despite CTS being an idiopathic syndrome, there are still existing risk factors associated with the prevalence of this medical condition. Notable ecological risk factors include extended positions in excesses of wrist flexion or extension, monotonous use of the flexor muscles, and exposure to vibration [15]. Unlike environmental factors, medical risk factors for CTS are classified into four categories. These include extrinsic factors, which increase the volume within the tunnel on either side of the nerve; intrinsic factors that increase the volume within the tunnel; extrinsic factors that alter the contour of the tunnel; and neuropathic factors [15-16]. Increasing rates of CTS events are also attributed to the increased life span for workers, as well as the increased cases of risk factors, such as diabetes and pregnancies. Extrinsic factors that increase the volume within the tunnel include circumstances that change the fluid equilibrium within the body. Such factors include pregnancy, menopause, obesity, kidney failure, hypothyroidism, use of oral contraceptives, and congestive heart failure. Intrinsic factors within the nerve for increasing the occupied volume inside the tunnel include lumps and tumor-like strains. These could be the outcomes of fractures of the distal radius, directly or through posttraumatic arthritis. Neuropathic factors include conditions such as diabetes, alcoholism, vitamin deficiency or toxicity, and exposure to toxins. These are significant factors since they affect the median nerve without necessarily increasing the interstitial pressure within the carpal tunnel. Diabetic patients have a higher propensity to develop CTS since they have a lower onset for nerve injury. In diabetic patients, the extent of incidence is 14% for patients without diabetes and 30% for patients with diabetic neuropathy, while the prevalence rate during pregnancy estimates at 2% [17].

Pathophysiology

The pathophysiology of CTS involves a combination of mechanical trauma, increased pressure, and ischemic damage to the median nerve within the carpal tunnel. Concerning increased pressure, normal pressure is recorded to vary between 2 mmHg and 10 mmHg. In the carpal tunnel, the change in the position of the wrist may result in dramatic shifts in the fluid pressure. As such, the extension increases the pressure to more than 10 times its initial level, while flexion of the wrist causes an eight times increase in the pressure [18]. Resultantly, repetitive motions in the wrist are significant risk factors for CTS incidences. In nerve injury, on the other hand, a noteworthy step in damage to the median nerve is demyelination, which occurs when the nerve is frequently exposed to automatic forces [19]. Demyelination of the nerve develops in the location of compression and spreads to the intermodal segment where the axons are left intact. With continuous compression, blood flow to the endoneurial capillary system is interrupted, causing alterations in the blood-nerve barrier and the development of endoneurial edema. As a result, a vigorous cycle begins, which consists of venous congestion, ischemia, and local metabolic alterations [18-19]. Ischemic injury is also noted as a significant element in CTS because of the assessment that symptoms rapidly resolve after carpal tunnel release surgery. Limb ischemia increases paraesthesias in carpal tunnel patients. This occurs in three phases, including increased intrafunicular pressure, injury to the capillary with leakage and edema, and obstruction of arterial flow in the patients [19].

Stages of CTS

In the first stage of the clinical diagnosis of CTS, the patient tends to wake up from sleep feeling numbness or swelling on the hand, with no noticeable swelling. The patient may feel extreme pain from the wrist spreading to the shoulder, with a tingling in the hand and fingers, which is defined as brachialgia paresthetica nocturna. On most occasions, the pain ceases after shaking the hand though the hand may feel firm later. The second stage of CTS development in the patient is the occurrence of symptoms, which occur in the day. Such symptoms occur when the patient engages in a repetitive activity involving the hand or wrist or if they maintain a specific position for extended periods [8,20]. Similarly, the patients may also note clumsiness when using their hands to grip objects, causing them to fall. The final stage of CTS development appears when there is hypotrophy or atrophy of the thenar eminence [20]. The occurrence of this stage also entails the ability to engage in any sensory symptoms by the patients.

Diagnostic tests

The diagnosis of CTS patients requires the respective medical professional to develop a case history associated with the characteristic signs of CTS. The patient should be questioned on the frequency of occurrence of these symptoms, whether they happen at night or during the day, or whether certain positions or repeated movements provoke the symptoms [8]. In addition, the doctor may question whether the patients use vibratory objects for their tasks, the parts of the arm where the sensations are felt, or if the patient may already have predisposing factors for CTS incidence. In this case, they may assess the patients for conditions associated with CTS such as diabetes, inflammatory arthritis, pregnancy, or hypothyroidism [21]. Physical assessment of the patient’s hand is a fundamental approach to the diagnosis of CTS since specific discoveries may indicate the availability of other factors. For instance, abrasions or ecchymosis on the wrist and hands may indicate that there has been damage to the tissue, which could also entail harm to the median nerve [22]. The initial medical tests for carpal tunnel syndrome are Tinel’s sign and Phalen’s maneuver. Tinel’s sign elicits a positive result when tapping over the along the carpal tunnel produces symptoms in the median nerve distribution. On the other hand, during Phalen’s maneuver, a patient flexes the wrist to 90 degrees, and the test is positive if the flexing produces symptoms along with the distribution of the median nerve. Additionally, monofilament testing, vibration, as well as two-point discrimination, could elicit sensory effects in carpal tunnel syndrome [22]. Using the patient’s medical history and physiological assessment may produce limited results and have less specific areas of symptom occurrence. Patients may, therefore, be required to complete a self-diagnosis questionnaire, described as the Katz Hand Diagram. This enables the patient to specify the parts of their hand that experience the symptoms and classify the symptoms like numbness, pain, tingling, or hypoesthesia [8].

Differential diagnosis 

During the diagnosis of CTS, it is essential to note that other conditions may also provide similar symptoms to CTS, thus requiring vigorous diagnosis to assert the medical condition of the patients. The differential diagnosis is essential when dealing with cases, such as the diagnosis of CTS in patients, by weighing the probability of one disease against other diseases that the patient could likely be suffering from. Thorough physiological assessment is an important strategy for a proper diagnosis to differentiate CTS from other health complications. The differential diagnosis distinguishes CTS from complications, such as carpometacarpal arthritis of the thumb, whose symptoms include excruciating thumb movement, positive grind evaluation, and radiographic outcomes [23]. Other conditions include cervical radiculopathy, whose symptoms include pain in the neck, numbness of the thumb and index finger, and positive results from the Spurling test; and de Quervain tendinopathy, which is responsible for tenderness at the distal radial styloid [22]. Others also include peripheral neuropathy, which shows a history of diabetes mellitus; pronator syndrome, whose symptoms include forearm pain, sensory loss over the thenar eminence, and weakness with thumb flexion, and wrist extension; and Raynaud syndrome, in which patients show symptoms associated with exposure to cold and typical change in color [22].

Management

The management of CTS incidences in patients depends on the severity of the disease. In minor and modest circumstances, a trial of conventional treatment is encouraged on the patients. This includes splinting, corticosteroids, physical therapy, therapeutic ultrasound, and yoga [22]. These forms of therapy encourage improved symptoms within two to six weeks, with the maximum benefit felt at three months. The use of splints is a significant response action for minor to moderate CTS due to its effortlessness, inexpensiveness, and admissibility [24]. It is also advisable for use in more reversible risk factors, such as pregnancy, and may be used to supplement other treatment approaches. Oral provision of prednisone at a 20 mg daily dose improves the symptoms and functions of the individual, as compared to the placebo, with its improvements lasting an average of eight weeks [22]. Another management option is engaging patients in physical therapy, including carpal bone mobilization, ultrasounds, and nerve glide exercises [24]. However, these tend to be less effective and require the presence of experienced therapists. On the other hand, patients suffering from severe CTS or nerve injury from electro-diagnostic results require surgical decompression as a method for the management of CTS [22]. Patients should be referred for surgical treatment if the symptoms persist, if there is no improvement in their health, or if the motor or sensory deficit is progressive [2,24].

Carpal tunnel release surgery

Over 80% of persons suffering from carpal tunnel syndrome have a positive response to conservative treatments. However, there is an 80% chance of the symptoms recurring in these patients within one year. Doctors should only consider surgery when the condition produces negative responses to conservative therapies. In essence, the objective of carpal tunnel release is to treat and hopefully relieve the patient from the painful experience resulting from carpal tunnel syndrome. Previously, physicians thought repetitive wrist and hand motions were the only cause of carpal tunnel syndrome, especially in frequent computer users. But now doctors understand that the syndrome is probably a congenital predisposition in that some individuals have bigger carpal tunnels as compared to others. Notably, injuries such as fracture or sprain and regular usage of vibrating equipment also cause carpal tunnel syndrome. In some cases, doctors link the syndrome to rheumatoid arthritis, diabetes, pregnancy, and thyroid disease. In words, carpal tunnel syndrome is a multifactorial condition. Carpal tunnel syndrome affects various parts of the wrist. The carpal tunnel forms a channel through which tendons and the median nerve pass through. The muscles and the median nerve facilitate the movement of fingers. The carpal tunnel comprises the wrist bones and the transverse carpal ligaments at the bottom and the top of the wrist [25]. Injuries or tightening of this part of the body causes the tissues in the tunnel to swell and compress the median nerve. When the pressing of the median nerve is not timely treated, it causes tingling and numbness on the hand, loss of function, and endless pain. While the symptoms begin slowly, they grow worse with time, and the pain is usually worse when the compression affects the thumb end of the wrist. During surgery, a surgeon often cuts through the pressing nerve, making incisions on the swollen parts. That creates extra room for the tendons and the median nerve passing through the carpal tunnel and often relieves pain and improves function. The only reason for surgery is the diagnosis of carpal tunnel syndrome. But even in such situations, doctors usually try the available non-surgical therapies first. The non-surgical treatment methods include physical therapy, wrist splints, medications, using alternative working tools at work, and the administration of steroids in the affected regions to ease the pain and swelling. The following are reasons that motivate doctors to recommend surgery often after the failure of non-surgical therapies. First, non-surgical treatments for carpal tunnel syndrome do not often relieve swelling and pain. Secondly, the surgeon conducts an electrophysiological examination of the median nerve and decides whether or not the patient has carpal tunnel syndrome. Third, the wrist and hand muscles are usually weak and usually get smaller due to severe compression of the median nerve. Lastly, doctors recommend surgery when the syndrome’s symptoms last more than six months without any relief [26]. In essence, non-surgical therapies would be the best option when one suspects they have carpal tunnel syndrome. The above reasons indicate that surgery could have more contraindications; hence, doctors do not recommend it first. Carpal tunnel release has several risks, similar to other operations. The wrist becomes numb, and the surgeon may administer local anesthesia to make the patient sleepy during the surgical procedure. In some instances, the doctors use general anesthesia to make the patient sleep deeply during the process. The use of anesthesia is contraindicative in some patients. Other probable risks associated with carpal tunnel surgery include infections, sensitive scars, bleeding, injuries to the nerves branching out from the median nerve, and injuries to neighboring blood vessels. The post-surgery recovery period ranges from a few weeks to many months. The recovery, in essence, depends on the extent and duration of the compression on the median nerve. Recovery procedures include wrist splinting and physical therapy to heal and strengthen the hand and the wrist. Doctors need to prepare a patient before surgery. Other medical conditions could lead to additional risks during and after the surgery. Hence, therefore, patients should discuss such pre-existing medical conditions. Also, the patient should tell the doctor about any medications they are using, including herbs, over-the-counter medicines, supplements, and vitamins [27]. In some cases, the physician recommends advises the patient to stop using the medications that would complicate the blood clotting process such as naproxen, aspirin, and ibuprofen [28]. It is also advisable for smokers to cease smoking before the carpal tunnel release because smoking delays healing. An electrocardiogram and blood tests are also essential before surgery. Lastly, the physician advises the patient not to drink and eat anything for up to 12 hours before the carpal tunnel surgery. The doctor could make further preparations based on the patient’s medical condition. Understanding the carpal tunnel release procedure is essential when addressing carpal tunnel syndrome. The surgery is often an outpatient process, meaning the patient can go home soon after surgery on the same day. There are two kinds of carpal tunnel release surgery, namely, the traditional approach and the endoscopic carpal tunnel release [8]. On the one hand, conventional medicine refers to the open release wherein the physician cuts open the wrist during the procedure. On the other hand, the endoscopic carpal tunnel release entails a thin and flexible tube containing a camera. The surgeon inserts the tube into the wrist via a small incision. The surgeon makes another cutting through which thin tools are inserted, and surgery proceeds following the guidance of the camera. Doctors use one of the types of carpal tunnel release surgeries, depending on the patient’s medical condition.

## Conclusions

CTS is a common medical condition that remains one of the most frequently reported forms of median nerve compression. CTS occurs when the median nerve is squeezed or compressed as it travels through the wrist. The syndrome is characterized by pain in the hand, numbness, and tingling in the distribution of the median nerve. This review of literature has provided an overview of CTS with an emphasis on anatomy, epidemiology, risk factors, pathophysiology, stages of CTS, diagnosis, and management options.
